# Adverse Birth Outcomes Associated with SARS-CoV-2 Infection Among Pregnant Women with and without HIV: A Longitudinal Cohort Study

**DOI:** 10.1097/QAI.0000000000003924

**Published:** 2026-11-01

**Authors:** Ibrahim Ahmed El-Imam, Abra Rachida Koudjra, Nginache Nampota-Nkomba, Felix Mkandawire, Osward Nyirenda, Rhoda Masonga, Cristiana Cairo, Melissa Gladstone, Miriam K. Laufer, Andrea G. Buchwald

**Affiliations:** Department of Epidemiology and Public Health, University of Maryland, Baltimore; Center for Vaccine Development and Global Health, University of Maryland School of Medicine; Blantyre Malaria Project, Kamuzu University of Health Sciences & University of Maryland, Baltimore; Blantyre Malaria Project, Kamuzu University of Health Sciences; Blantyre Malaria Project, Kamuzu University of Health Sciences; Blantyre Malaria Project, Kamuzu University of Health Sciences; University of Maryland, Baltimore; University of Liverpool; Center for Vaccine Development and Global Health, University of Maryland School of Medicine; Center for Vaccine Development and Global Health, University of Maryland School of Medicine; Department of Clinical Sciences, Liverpool School of Tropical Medicine

**Keywords:** Sars-CoV-2, HIV, pregnancy, pregnancy outcomes, low birth weight, small for gestational age

## Abstract

**Background:**

COVID-19 and HIV infection are independently associated with increased risk of adverse pregnancy outcomes. However, there is limited evidence on the impact of SARS-CoV-2 infection during pregnancy in sub-Saharan Africa, particularly among women living with HIV (WLWH).

**Setting:**

Two antenatal clinics in southern Malawi (2018 – 2022).

**Methods:**

We conducted a prospective cohort study of singleton pregnancies enrolled at 20–36 weeks gestation. SARS-CoV-2 infection was determined via serologic testing at enrollment and delivery. Participants were enrolled based on HIV status and viral suppression: (1)WLWH with detectable viral load (VL), (2)WLWH with undetectable VL, and (3)HIV-negative women. We used multivariable logistic regression with adjustment for confounding to evaluate the impact of SARS-CoV-2 alone or in combination with HIV infection on the following adverse birth outcomes: low birth weight (LBW), preterm birth, small-for-gestational-age (SGA), and stillbirth or early neonatal death.

**Results:**

Among 905 pregnant women, 29% tested positive for SARS-CoV-2, 87% of infections were mild or asymptomatic. Overall, SARS-CoV-2 infection was significantly associated with SGA births (adjusted OR [aOR]: 1.49, 95% CI: 1.03–2.13) but not associated with other adverse outcomes. Among WLWH, SARS-CoV-2 was significantly associated with increased odds of LBW (aOR: 2.07, 1.10–3.91) and SGA births (aOR: 1.73, 1.01 – 2.91). The effect of SARS-CoV-2 infection among WLWH did not differ based on VL.

**Conclusion:**

SARS-CoV-2 infection during pregnancy was associated with adverse birth outcomes, particularly among WLWH. These findings underscore the need for integrated antenatal care and targeted infection prevention strategies for pregnant women with HIV in high-burden settings.

## Introduction

The COVID-19 pandemic, caused by the severe acute respiratory syndrome coronavirus 2 (SARS-CoV-2), continues to pose a significant global public health challenge, especially among vulnerable groups. While people of all ages are susceptible to infection, pregnant women and people living with human immune-deficiency virus (HIV) infection are at increased risk of severe illness and mortality due to COVID-19 (1–4). Pregnant women are particularly at increased risk of complications from SARS-CoV-2 due to physiological changes that occur during pregnancy (5). SARS-CoV-2 infection in pregnancy has been associated with severe adverse maternal and perinatal outcomes such as increased maternal hospitalization, preterm births, small for gestational age, and low birth weight (6–10). Similarly, HIV infection significantly increases the risk of adverse maternal and perinatal outcomes among pregnant women living with HIV (WLWH) (11–14), particularly among individuals with high HIV viral load (15,16).

Sub-Saharan Africa (SSA) remains the epicenter of the global HIV/AIDS epidemic, with the majority of infections found in women (17). In Malawi, HIV prevalence is estimated at 8.9% among adults aged 15–64 years, and 10.5% among women (18). Estimates of SARS-CoV-2 seroprevalence in Malawi throughout the pandemic ranged from 8% in late 2020 (19), to 89% in rural areas and 94% in urban settings by April 2022 (20), with all studies reporting largely asymptomatic transmission. The high prevalence of HIV in Malawi, as in other SSA countries, may exacerbate the risk of morbidity and mortality related to SARS-CoV-2, particularly among pregnant women (6,21). WLWH may also experience an increased disease burden with SARS-CoV-2 compared to those without HIV infection. This is of particular concern among individuals with high viral loads and low CD4 counts, who show delayed antibody responses against SARS-CoV-2 (10,21–26). This situation raises concerns about the combined effects of HIV and SARS-CoV-2 infections on pregnancy outcomes.

There is extremely limited data on the effect of SARS-CoV-2 infections among pregnant women in SSA or among WLWH. One previous study in Botswana found that HIV and SARS-CoV-2 co-infections interacted to elevate the risk of adverse pregnancy outcomes (21). However, there is a critical need for more research focused on understanding the combined effects of HIV and SARS-CoV-2 co-infection on pregnant women, particularly as no studies have examined how the impact of SARS-CoV-2 infection varies by HIV viral load or ART exposure. The burden of the COVID-19 pandemic is likely underestimated due to a lack of data in these high-risk groups, but understanding the impact of SARS-CoV-2 on pregnant WLWH can both help to inform current SARS-CoV-2 response and future pandemic preparedness. This study aims to evaluate the impact of SARS-CoV-2 exposure on adverse pregnancy outcomes by HIV status and will further investigate the role of HIV viral load in modifying the impact of SARS-CoV-2 infection on these outcomes. We hypothesize that SARS-CoV-2 infection will significantly worsen the risk of adverse pregnancy outcomes, with more severe effects anticipated among pregnant WLWH, particularly those with high viral loads, compared to their virally suppressed or HIV-negative counterparts. This research seeks to fill critical gaps in our understanding of the combined effects of HIV and SARS-CoV-2 infections on pregnancy outcomes in SSA.

## Methods

### Study Design and population.

We conducted a prospective cohort study among pregnant women in three cohorts based on their HIV and viral load status in two research clinics near Blantyre, Malawi. Cohort recruitment has been described previously (27). The study enrolled pregnant women at antenatal care (ANC) clinics in their second trimester and followed them through delivery, with their newborn infants monitored postnatally. Pregnant women were recruited into three cohorts according to their HIV status and viral load: 1) Pregnant WLWH with high viral loads (>10,000 copies/ml), with no previous reported ART use; 2) Pregnant WLWH with undetectable viral loads (<400 copies/ml), reporting ongoing ART use; and 3) Pregnant women uninfected with HIV. Pregnant women were enrolled between 2018 and 2022, spanning both the pre-pandemic and COVID-19 pandemic period. Malawi reported its first confirmed COVID-19 case in April 2020 and subsequently experienced multiple epidemic waves associated with Beta, Delta, and Omicron variants despite limited testing and genomic surveillance capacity throughout Malawi (28,29).

### Study setting and eligibility.

Participants were recruited from two ANC clinics - Ndirande and Bvumbwe. Ndirande Health Facility is located in the urban area of Ndirande, Blantyre, Malawi. It serves a densely populated community, providing a wide range of health services including maternal and child health, outpatient care, and HIV testing and treatment. Bvumbwe Health Facility is situated in the rural area of Bvumbwe, Thyolo District, Malawi. It serves a largely agrarian community, offering essential health services such as antenatal and postnatal care, HIV testing and treatment, immunizations, and treatment for common illnesses. SARS-CoV-2 vaccination and testing was not widely available in Malawi at the time of the study with only 4% of eligible adults vaccinated at the time recruitment ended (30), making it unlikely that any participants were vaccinated.

Eligible participants were any pregnant women aged 18 years and older, who attended their first ANC visit between 20–36 weeks gestation (confirmed by ultrasound) at one of the study clinics, intended to breastfeed, were willing to deliver at the clinic, and planning to remain in the study area for at least 1 year. HIV-negative women were required to test negative by rapid HIV test at both enrollment and delivery. We excluded women with multiple gestations. All WLWH were on ART and monitored for ART adherence for the duration of the study and no opportunistic infections were detected during the study. All women with samples at enrollment and delivery for retrospective SARS-CoV-2 antibody assessment were included in this analysis, so some women were enrolled and delivered before the pandemic began and did not have opportunity to become infected with SARS-CoV-2. There were decreased health services available from April to June 2020, and women who delivered during this time did not provide samples and could not be included in retrospective SARS-CoV-2 serological assessment or this analysis.

### Data collection

Eligible participants underwent initial screening including HIV testing, documentation of ART exposure, gestational age assessment via ultrasound, and baseline viral load measurements for participants with HIV. Serum samples were collected at enrollment and at delivery from all participants and assessed for anti-SARS-CoV-2 antibodies retrospectively. Samples were assessed for anti-SARS-CoV-2 IgM and IgG using the RayBiotech COVID-19 S1 RBD Protein Human IgG ELISA Kit, which provides quantitative measurements of IgG antibodies and the Inbios SCoV-2 Detect IgM ELISA Kit, a qualitative test that detects the presence or absence of IgM antibodies. The selection of the S1 RBD is based on its high specificity for SARS-CoV-2, as pre-pandemic studies show minimal cross-reactivity with this protein, thereby minimizing false positives and enhancing the accuracy of infection identification (31).

### Variables and definitions

#### Exposure

Exposure in this study is SARS-COV-2 infection during pregnancy determined through serological testing for IgM and IgG antibodies. Participants were classified as infected during pregnancy if their serological testing met one of two criteria; 1) Positive for anti-SARS-CoV-2 IgM antibodies at either the screening or delivery visit because IgM antibodies against SARS-COV-2 typically appear within the first few weeks of infection and persist for several weeks (31,32) or 2) if participants seroconverted by IgG from negative at screening to positive at delivery or if the concentration of IgG antibodies at delivery was at least 3.5-fold higher than the concentration at screening. We did not consider IgG positive samples at screening as evidence of recent infection as IgG persists for up to nine to twelve months after exposure (33). The precise timing of infection during pregnancy and whether it represented a first infection could not be determined.

### Primary outcome

The primary outcome of this study is any adverse birth outcome, which covers the occurrence of any of the following - maternal hospitalization, early neonatal death (ENND) (34), stillbirth (35), low birth weight (LBW) (36), small for gestational age (SGA) (37), and preterm birth (38). Additional other outcomes include: 1) LBW, 2) Preterm birth, 3) SGA birth, and 4) the combined outcome of ENND or stillbirth. According to the World Health organization (WHO) ENND is defined as a newborn death of a live-born baby within 7 days of life, and stillbirth as the death of a fetus before birth, which includes both antenatal and intrapartum deaths (34,35). Preterm birth is defined as birth that occurs before 37 weeks of gestation. LBW is defined as birth weight less than 2500 grams (up to and including 2499 grams), and SGA is defined as newborns with birth weight which is less than the 10th percentile for their gestational age (36–38). Outcomes are not mutually exclusive and pregnancies may have qualified for more than one adverse outcome, i.e. LBW births are often either preterm or SGA. Adverse outcomes in this cohort have previously been described at length (27).

### Covariates

We selected covariates for final adjusted models using a Directed Acyclic Graph (Supplemental Figure 1). We categorized variables into 1) minimally sufficient confounders, identified *a priori* including: gravidity (primigravida/multigravida), maternal age (continuous variable in years), and residential location of each clinic (rural Bvumbwe/urban Ndirande) 2) Effect modifiers, assessed for interaction including: HIV status (positive/negative) and viral load status among WLWH (detectable/undetectable at screening and delivery), and CD4 count, (<350 cells/mL/≥350 cells/mL, and 3) Descriptive covariates, used in preliminary analysis to describe the study population but were not adjusted for in the final regression models including: Education level, ART regimen type (non-nucleoside reverse transcriptase inhibitor- [NNRTI-based]/ Dolutegravir [DTG-based]), and marital status (married or unmarried). Pre-selected confounders were removed from final adjusted models if they did not improve the model fit or alter the estimate of the main effect.

### Data analysis

We used univariate analyses to compute frequencies and proportions of participant characteristics at baseline, describing continuous variables with means and standard deviations and categorical variables with proportions. Bivariate relationships between participants’ sociodemographic and clinical characteristics at baseline and SARS-CoV-2 status were examined using chi-squared tests for categorical variables and Student’s t-tests for continuous variables, with a significance level set at α=0.05 to determine differences in SARS-CoV-2 seroconversion. We also assessed the bivariate relationship between baseline demographic characteristics and each outcome variable to identify significant predictors.

We employed logistic regression to explore the association between SARS-CoV-2 seropositivity and four pregnancy outcomes: low birthweight, preterm birth, small-for-gestational-age, and the composite ‘any adverse outcome’. Due to small numbers of stillbirths and ENND included in this study, we were unable to assess them as an outcome in regression models. Crude odds ratios (OR) and 95% confidence intervals (CI) were calculated to test these associations. To examine the combined risk of SARS-CoV-2 seropositivity and HIV on pregnancy outcomes, we evaluated an interaction term (SARS-CoV-2*HIV) in the models. Additionally, we conducted stratified analyses for SARS-CoV-2 infection by HIV and viral load status to explore the impact of viral load on each adverse pregnancy outcome. While CD4 levels were initially considered as an effect modifier, low power in sub-analysis (due to small cell counts) prevented meaningful interpretation. In the final adjusted multivariable logistic regression model, we adjusted for facility location (site) and gravidity.

We conducted a sensitivity analysis, repeating all analysis in a restricted analytic cohort of women enrolled on or after April 1, 2020, to address differences in SARS-CoV-2 exposure opportunity due to differential recruitment timing between WLWH and HIV-negative women.

### Sample size and power

With a sample size of 905 pregnant women, including 258 exposed to SARS-CoV-2, assuming 23% prevalence of adverse pregnancy outcomes, our study has an 86.2% power to detect a 10% difference in adverse birth outcomes between SARS-CoV-2 positive and negative groups, using a two-sided hypothesis with a 0.05 significance level.

## Results

### Characteristics of the study population

The initial study recruited 1,781 pregnant women in their second trimester of pregnancy, of whom 905 were followed through to delivery, had samples tested for SARS-CoV-2 and were included in this analysis. (Figure 1). Among these participants, 29% tested positive for SARS-CoV-2 (n = 258). The study participants were evenly distributed between the two sites with a mean maternal age of 28 years. Among women who tested positive for SARS-CoV-2, 60% were HIV-negative and 40% were WLWH (p<0.001)) (Table 1). A greater proportion of WLWH than those without HIV were recruited into the cohort before the onset of the COVID-19 pandemic. Among the 184 women enrolled before 2020 who were tested for SARS-Cov-2, 147 (80%) were WLWH. This differential timing of recruitment likely contributed to the observed difference in SARS-CoV-2 positivity between the two groups.

### SARS-CoV-2 Symptoms and Severity

Among the 905 women tested for SARS-CoV-2, 401 (44%) sought treatment during follow-up for illness or injury. Among women with SARS-CoV-2 infections, only 34/258 (13%) sought treatment for respiratory symptoms during pregnancy. Those that sought treatment with respiratory symptoms additionally reported muscle aches (n = 11, 32%), headache (n = 10, 29%), and fever (n = 7, 20%), with four (12%) women reporting chest pain or shortness of breath. Two SARS-CoV-2 positive women were diagnosed with pneumonia.

### Pregnancy Outcomes:

Among 905 women, 297 (33%) experienced any adverse pregnancy outcome. The most frequent outcome was SGA birth (168 cases, 19%), followed by LBW (80 cases, 9.1%), and preterm birth (79 cases, 8.7%). Among LBW births, 95% were classified as either preterm or SGA as well. A total of 13 stillbirths and three early neonatal deaths were recorded, with only two stillbirths occurring among women with SARS-CoV-2–positive pregnancies. SARS-CoV-2 infection was significantly associated with SGA in preliminary analysis with 59 (24%) of SARS-CoV-2 infected women having SGA births, compared to 109 (17%) of women without SARS-CoV-2 infections. However, SARS-CoV-2 was not significantly associated with LBW, preterm birth, or early neonatal death in the total population (Table 2).

### Association Between Adverse Pregnancy Outcomes and Covariates

In bivariate analysis (Supplemental Tables 1a–1d), we assessed the relationship between baseline sociodemographic characteristics and each of the five adverse pregnancy outcomes. We found that LBW was significantly associated with HIV status, site, gravidity, and parity, while other adverse pregnancy outcomes, including the composite outcome of any adverse events, preterm, SGA birth, and stillbirth/early neonatal death showed associations with sociodemographic factors such as gravidity, parity, maternal age, site, delivery method, and HIV status.

### Regression Models for Impact of SARS-CoV-2 Infection on Pregnancy Outcomes

Among all women in the study, regardless of HIV status, we assessed the unadjusted association between SARS-CoV-2 and each of the adverse pregnancy outcomes. Only SGA showed a statistically significant association with SARS-CoV-2 positivity in the total population (OR: 1.48, 95% CI: 1.03–2.11, p=0.03) (Table 3, Figure 2). Stratified analysis by HIV status found no evidence of association between SARS-CoV-2 and adverse pregnancy outcomes among HIV-negative pregnant women (Figure 2). However, among WLWH, SARS-CoV-2 positivity was significantly associated with an increased odds of LBW (aOR of 2.04 (95% CI: 1.09–3.82, p = 0.023), and SGA birth (aOR = 1.73. 95% CI: 1.01 – 2.91, p = 0.04) (Figure 2). SARS-CoV-2 positivity was not associated with preterm birth or stillbirth/early neonatal death in either group. There was significant interaction between HIV status and SARS-CoV-2 infection in models for LBW, but not for any of the other outcomes, and final adjusted models are shown in Table 3. Final multivariable regression models (Table 3) were adjusted for site and gravidity, as these were identified as key confounders from our directed acyclic graph and preliminary analysis.

### Impact of Viral Load on SARS-CoV-2 and Pregnancy Outcomes

To assess whether HIV viral load modifies the effect of SARS-CoV-2 on pregnancy outcomes, we conducted a stratified analysis among WLWH with detectable and undetectable viral load (Table 4). There was no statistically significant association between SARS-CoV-2 and any adverse outcome across viral load status due to lower power in the HIV sub-groups. However, the magnitude and direction of effect for all outcomes was similar by viral load detection status. In a sensitivity analysis restricted to women enrolled on or after April 1, 2020 (N=709), the direction and magnitude of effect estimates were consistent with the primary analysis across all outcomes and subgroups. The association between SARS-CoV-2 infection and SGA in the total population (aOR:1.38, 95% CI:0.93 – 2.05) and LBW among WLWH (aOR: 1.53, 95% CI: 0.73 – 3.13) were attenuated and no longer statistically significant. Full sensitivity analysis results are presented in Supplemental Tables 2a – 2d.

## Discussion

In this cohort of Malawian pregnant women, serological evidence of SARS-CoV-2 infection during pregnancy was significantly associated with an increased risk of SGA birth in all included pregnant women, regardless of HIV status. Notably, among WLWH, SARS-CoV-2 infection was significantly associated with an increased risk of both LBW and SGA. These associations did not differ significantly regardless of whether or not the woman had fully controlled HIV (undetectable viral load) throughout pregnancy, although power was limited for these sub-analyses. These findings indicate that SARS-CoV-2 seropositivity during pregnancy, even in the absence of severe COVID-19 disease, is associated with increased risk of adverse pregnancy outcomes, and WLWH are particularly vulnerable to the adverse effects of SARS-CoV-2 infection.

This is one of the first reports of the impact of SARS-CoV-2 infection on adverse birth outcomes in SSA, and our findings are consistent with previous studies conducted in high-income settings (7,8,10,26,39). A systematic review of 42 studies found SARS-CoV-2 infection associated with increased risk of preterm birth (OR 1.82; 95% CI 1.38, 2.39), stillbirth (OR 2.11; 95% CI 1.14, 3.9), and low birth weight (OR 1.89; 95% CI 1.14, 3.12)(6). Our study was designed before the COVID-19 pandemic began and did not include surveillance for clinical COVID-19 disease. Few participants sought care for respiratory symptoms during the study, and as such, many of the infections we identified were likely mild or asymptomatic. Previous studies have found increasing severity of COVID-19 symptoms in pregnant women is associated with higher risk of adverse outcomes (6). The mild nature of most infections in our study may have contributed to the less severe effect of SARS-CoV-2 in our overall study population, and particularly among healthy women without HIV, compared to previous studies. The absence of active disease surveillance limits our ability to fully characterize the potential impact of COVID-19 disease. However, our findings of increased risk of adverse pregnancy outcomes among a cohort with mostly mild infections are important, indicating that even mild or asymptomatic SARS-CoV-2 infections may influence pregnancy outcomes, particularly among vulnerable populations, highlighting the importance of infection prevention among WLWH and other high-risk populations.

Previous studies from sub-Saharan African nations examining the impact of SARS-CoV-2 infection on adverse birth outcomes in the presence of other comorbidities are few (6) and frequently do not include controls without COVID-19, making them difficult to interpret. Our findings are in agreement with one previous study examining the combined effect of HIV and SARS-CoV-2 coinfection in Botswana, which reported increased risk of preterm birth and stillbirth among pregnant women with SARS-CoV-2 infection, with the greatest burden observed among WLWH (21). In contrast, recent data from Botswana during the Omicron era reported no significant association between SARS-COV-2 infection and adverse birth outcomes, including among WLWH (40), suggesting any effect of Sars-CoV-2 on adverse pregnancy outcomes may be variant specific. Continuous monitoring and further research measuring coronavirus cases and other respiratory infections in pregnant women in Sub-Saharan Africa are necessary to fully understand the risks of SARS-CoV-2 infections and future pandemic respiratory infections on pregnant women in Sub-Saharan Africa (26).

Despite the low overall risk of adverse outcomes related to SARS-CoV-2 in our study population, among WLWH, there was a two-fold significantly higher risk for LBW and 73% higher odds of SGA birth as a result of SARS-CoV-2 exposure. This finding did not differ by HIV viral load status; however, the power of this sub-analysis was low as there were few women with detectable viral load. Although CD4 count data were available for a subset of participants, substantial missingness precluded a reliable assessment of the impact of CD4-defined immunosuppression. These findings may be driven by HIV-related immune dysregulation and chronic inflammation, rather than viral load differences. Studies have shown that HIV infection induces persistent immune activation, systemic inflammation, and placental insufficiency, all of which can impair fetal growth increasing the odds of LBW and SGA births (15,41–43). Similarly, SARS-CoV-2 infection has been reported to cause vascular dysfunction and placental inflammation (44,45). Thus, co-infection with HIV and SARS-CoV-2 infections may have a synergistic effect, exacerbating placental dysfunction. Alrubayyi et al. reported that even with undetectable viral load immune dysfunction persists, affecting adaptive response to SARS-CoV-2 (23). Our findings further emphasize the consequences of this immune dysfunction and the importance of prioritizing vaccination and other preventive services for WLWH.

Among the women included in our analysis, 29% tested positive for SARS-CoV-2, with 60% of these being HIV-negative. The proportion of SARS-CoV-2 positivity was higher among HIV-negative pregnant women compared to WLWH (33.7% vs. 23.2%). This disparity likely reflects difference in timing of recruitment and biological response. Notably, 36% of the WLWH were recruited before Malawi’s first confirmed COVID-19 case, compared to only 1% of HIV negative women. As such, a larger proportion of the HIV negative women were recruited during periods of high SARS-CoV-2 community transmission. To assess whether differential recruitment timing influenced our findings, we conducted a sensitivity analysis restricted to women enrolled after April 2020. The direction and magnitude of effects estimates were consistent across all outcomes and subgroups; however, power was limited to detect statistically significant differences in this restricted cohort. Additionally, prior studies suggest that people living with HIV may mount a dampened serological response to SARS-CoV-2 (23) potentially leading to misclassification of the exposure with a resultant underestimation of true effect among WLWH, biasing results towards the null. Furthermore, we did not perform PCR testing at the time of illness, which would have been necessary to confirm symptomatic COVID-19. As a result, many of the infections we detected in the cohort were likely asymptomatic or mild enough that participants did not seek care. Participant-level viral sequencing data were unavailable preventing assessment of differences by viral variants. Despite these limitations, we found a significant effect of SARS-CoV-2 infection on adverse pregnancy outcomes, indicating that the true effect of SARS-CoV-2 on pregnancy outcomes may be greater than estimated in this study, particularly among WLWH.

This study benefits from a prospective cohort study design, allowing for the observation of pregnancy outcomes over time and providing robust evidence for discerning the effects of SARS-CoV-2 infection on pregnancy outcomes. The design enabled sub-analyses by HIV and viral load status, highlighting the differential impacts of these factors within an underrepresented setting in current literature.

In conclusion, our study provides additional evidence of the increased risk pregnant women face as a result of SARS-CoV-2 infection, regardless of clinical symptoms, and in particular among WLWH. We additionally found evidence that viral suppression alone is insufficient to prevent the increased risk due to SARS-CoV-2 infection among WLWH. These findings may help inform public health policies and interventions aimed at improving maternal and child health outcomes, emphasizing the importance of integrated care models, ongoing surveillance, and tailored preventive services in the context of viral pandemics, particularly among WLWH. We recommend enhanced prenatal care including counseling on infection prevention measures and prioritized vaccination for pregnant WLWH.

## Supplementary Material

supplemental figures and tables

supplemental----http://links.lww.com/QAI/C715

## Figures and Tables

**Figure 1:**
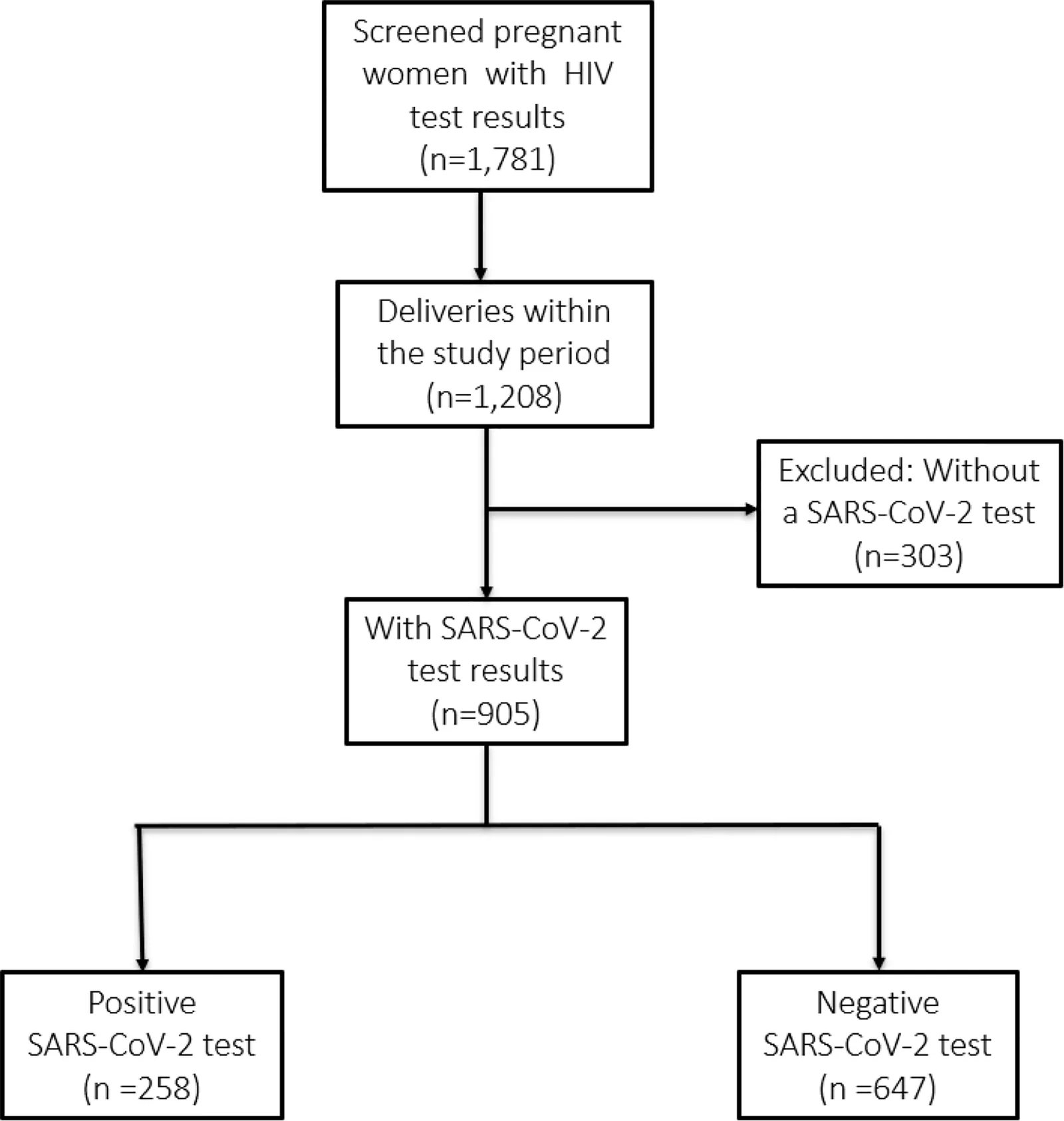
Study Flow Diagram Abbreviations: HIV, human immunodeficiency virus; SARS-CoV-2, severe acute respiratory syndrome coronavirus-2

**Figure 2:**
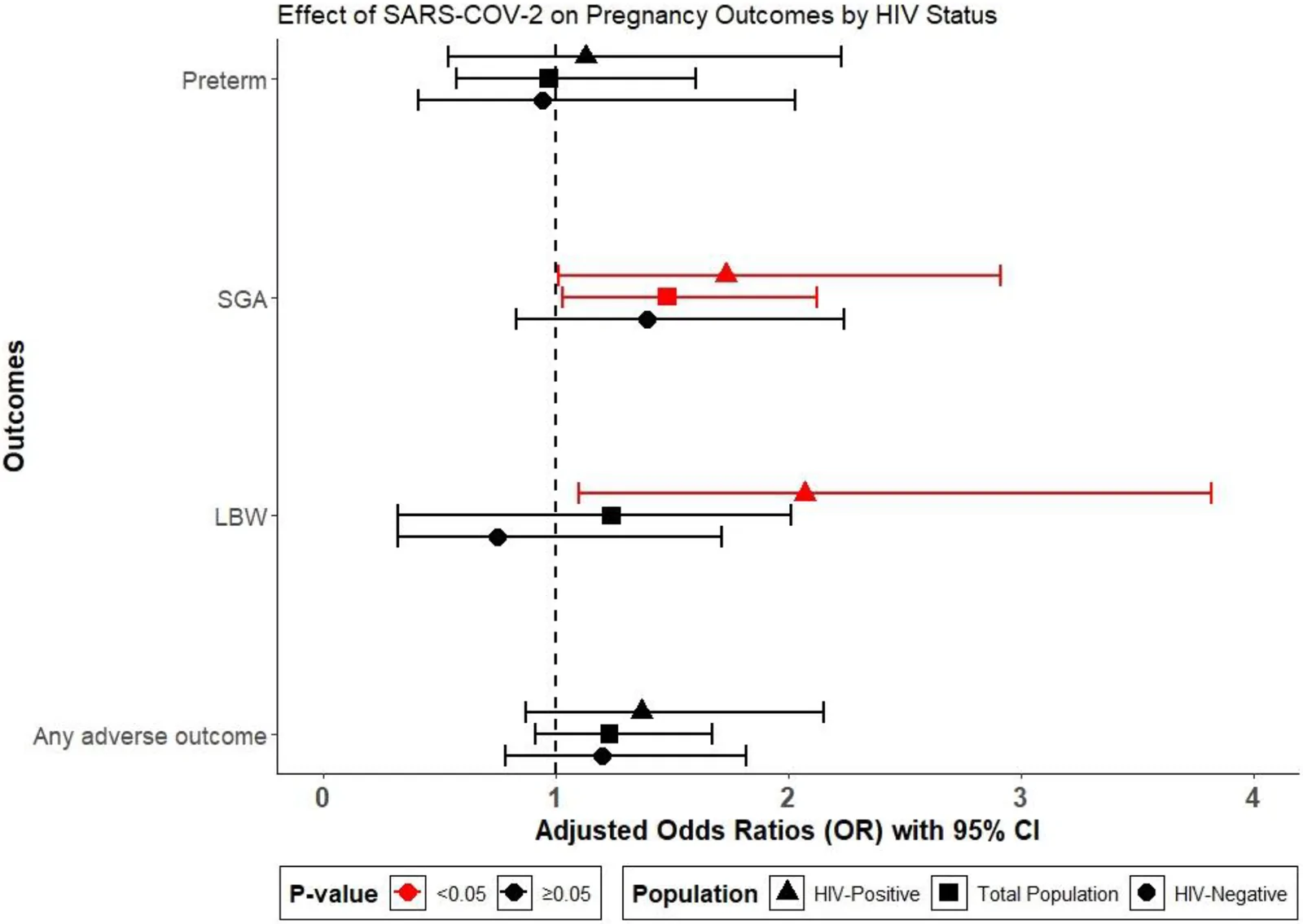
Adjusted Odds Ratios from logistic regression for effect of SARS-CoV-2 infection compared to pregnant women without SARS-CoV-2 infections. Models adjusted for Site and Gravidity. Abbreviation: SGA= small-for-gestational-age, LBW= low birth weight

**Table 1:** Characteristics of study participants by SARS-COV-2 Exposure

Characteristic	Overall^1^ N=905	SARS-CoV-2 Negative^1^ N =647	SARS-CoV-2 Positive^1^ N = 258	p-value^2^
**Site**				
Bvumbwe	412 (46%)	293 (45%)	119 (46%)	0.8
Ndirande	493 (54%)	354 (55%)	139 (54%)	
**Maternal Age**				
Mean (SD)	28 (6)	28 (6)	28 (7)	0.1
**Marital Status**				
Married	819 (91%)	587 (91%)	232 (90%)	0.8
Not Married	85 (9%)	60 (9%)	25 (10%)	
**Educational Level**				
Below Secondary	461 (51%)	336 (52%)	125 (49%)	0.4
Secondary and above	443 (49%)	311 (48%)	132 (51%)	
**Gravidity**				
Multigravida	785 (87%)	564 (87%)	221 (86%)	0.5
Primigravida	120 (13%)	83 (13%)	37 (14%)	
**Delivery Method**				
Others	61 (7%)	46 (7%)	15 (6%)	0.5
Vaginal	844 (93%)	601 (93%)	243 (94%)	
**Infant Sex**				
Female	444 (49%)	318 (49%)	126 (49%)	>0.9
Male	461 (51%)	329 (51%)	132 (51%)	
**HIV Status**				
Negative	457 (50%)	303 (47%)	154 (60%)	**<0.001**
Positive	448 (50%)	344 (53%)	104 (40%)	
**CD4 Counts**				
Mean (SD)	589 (242)	577 (237)	630 (255)	0.07
**Viral Load**				
Detectable	72 (16%)	55 (16%)	17 (16%)	>0.9
Undetectable	376 (84%)	289 (84%)	87 (84%)	

1 n (%);

2 Pearson’s Chi-squared tests for categorical variables and Wilcoxon rank sum test for continuous variables; SD = standard deviation;

**Table 2:** Pregnancy Outcomes by SARS-CoV-2 Exposure

Characteristic	Overall N = 905^1^	SARS-COV-2 Negative N = 647^1^	SARS-COV-2 Positive N = 258^1^	p-value^2^
Any adverse outcome				
Present	297 (33%)	204 (32%)	93 (36%)	0.2
Absent	608 (67%)	440 (68%)	164 (64%)	
Low Birth Weight				
Present	80 (9%)	54 (8%)	26 (10%)	0.4
Absent	798 (91%)	575 (91%)	223 (90%)	
Small for Gestational Age				
Absent	709 (81%)	519 (83%)	190 (76%)	**0.03**
Present	168 (19%)	109 (17%)	59 (24%)	
Hospitalization				
Present	75 (8%)	56 (9%)	19 (7%)	0.5
Absent	829 (92%)	590 (91%)	239 (93%)	
Preterm Birth				
Present	79 (9%)	57 (9%)	22 (9%)	0.9
Absent	826 (91%)	590 (91%)	236 (91%)	
Early neonatal death/stillbirth				
Present	16 (2%)	14 (2%)	2 (1%)	0.4
Absent	889 (98%)	633 (98%)	256 (99%)	

1 n (%),

2 Pearson’s Chi-squared test

**Table 3:** Association between SARS-COV-2 Exposure and Pregnancy Outcomes from Logistic Regression Models

Outcomes		SARS-CoV-2		Unadjusted			Adjusted		
	Overall	Negative	Positive	OR	95% CI	p-value	aOR	95% CI	p-value
**Total Population**									
**Any Adverse Event**									
Present	297 (33%)	204 (32%)	93 (36%)	1.22	0.90, 1.66	0.2	1.23	0.91, 1.67	0.2
Absent	608 (67%)	440 (68%)	164 (64%)						
**Low Birth Weight**									
Present	80 (9.1%)	54 (8.6%)	26 (10%)	1.24	0.75,2.01	0.4	1.24	0.75, 2.02	0.4
Absent	798 (91%)	575 (91%)	223 (90%)						
**Preterm Birth**									
Present	79 (8.7%)	57 (8.8%)	22 (8.5%)	0.96	0.57,1.59	0.9	0.97	0.57, 1.60	0.9
Absent	826 (91%)	590 (91%)	236 (91%)						
**Small for Gestational Age**									
Present	168 (19%)	109 (17%)	59 (24%)	**1.48**	**1.03, 2.11**	**0.03**	**1.49**	**1.03, 2.13**	**0.03**
Absent	709 (81%)	519 (83%)	190 (76%)						
**Women without HIV**									
**Any Adverse Event**									
Present	137 (30%)	87 (29%)	50 (32%)	1.27	0.94, 1.73	0.12	1.2	0.78, 1.82	0.4
Absent	320 (70%)	216 (71%)	104 (68%)						
**Low Birth Weight**									
Present	29 (6.4%)	21 (7.0%)	8 (5.3%)	0.74	0.32, 1.71	0.5	0.76	0.31, 1,70	0.5
Absent	423 (94%)	279 (93%)	144 (95%)						
**Preterm Birth**									
Present	31 (6.8%)	21 (6.9%)	10 (6.5%)	0.93	0.43, 2.03	0.86	0.94	0.41, 2.00	0.9
Absent	426 (93%)	282 (93%)	144 (94%)						
**Small for Gestational Age**									
Present	81 (18%)	49 (16%)	32 (21%)	1.37	0.83, 2.24	0.2	1.39	0.83, 2.29	0.2
Absent	371 (82%)	251 (84%)	120 (79%)						
**Women Living with HIV**									
**Any Adverse Event**									
Present	284 (63%)	224 (65%)	60 (58%)	1.37	0.87, 2.15	0.2	1.37	0.87, 2.15	0.2
Absent	164 (37%)	120 (35%)	44 (42%)						
**Low Birth Weight**									
Present	51 (12%)	33 (10%)	18 (19%)	**2.04**	**1.09, 3.82**	**0.03**	**2.07**	**1.10, 3.91**	**0.03**
Absent	375 (88%)	296 (90%)	79 (81%)						
**Preterm Birth**									
Present	48 (11%)	36 (10%)	12 (12%)	1.13	0.56, 2.23	5	1.13	0.54, 2.21	0.7
Absent	400 (89%)	308 (90%)	92 (88%)						
**Small for Gestational Age**									
Present	87 (20%)	60 (18%)	27 (28%)	**1.72**	**1.02, 2.91**	**0.04**	**1.73**	**1.01, 2.91**	**0.04**
Absent	338 (80%)	268 (82%)	70 (72%)						

OR = Odds Ratio, aOR = adjusted Odds Ratio, adjusted models adjusted for site and gravidity

**Table 4:** Unadjusted association between SARS-CoV-2 Infection and Pregnancy Outcomes Among Women Living with HIV by Viral Load Status

All HIV Positive	Overall*^1^*	SARS-CoV-2 Negative*^1^*	SARS-CoV-2 Positive*^1^*	OR	95% CI	p-value*^2^*
**Any Adverse**						
**Event**						
Present	164 (37%)	120 (35%)	44 (42%)	1.37	0.87, 2.14	0.2
Absent	284 (63%)	224 (65%)	60 (58%)			
**Low Birth Weight**						
Present	51 (12%)	33 (10%)	18 (19%)	**2.04**	**1.08, 3.78**	**0.02**
Absent	375 (88%)	296 (90%)	79 (81%)			
**Preterm Birth**						
Present	48 (11%)	36 (10%)	12 (12%)	1.12	0.54, 2.18	0.8
Absent	400 (89%)	308 (90%)	92 (88%)			
**SGA**						
Present	87 (20%)	60 (18%)	27 (28%)	**1.72**	**1.01, 2.89**	**0.04**
Absent	338 (80%)	268 (82%)	70 (72%)			
**Detectable viral load**						
**Any Adverse**						
**Event**						
Present	31 (36%)	24 (35%)	7 (41%)	1.5	0.90, 2.49	0.6
Absent	55 (64%)	45 (65%)	10 (59%)			
**Low Birth Weight**						
Present	7 (8.6%)	5 (7.5%)	2 (14%)	2.07	0.36, 11.92	0.4
Absent	74 (91%)	62 (93%)	12 (86%)			
**Preterm Birth**						
Present	8 (9.3%)	6 (8.7%)	2 (12%)	1.3	0.55, 2.84	0.7
Absent	78 (91%)	63 (91%)	15 (88%)			
**SGA**						
Present	16 (20%)	12 (18%)	4 (29%)	1.6	0.87, 2.86	0.5
Absent	65 (80%)	55 (82%)	10 (71%)			
**Undetectable viral load**						
**Any Adverse**						
**Event**						
Present	119 (34%)	85 (32%)	34 (41%)	1.23	0.40, 3.62	0.1
Absent	228 (66%)	180 (68%)	48 (59%)			
**Low Birth Weight**						
Present	40 (12%)	26 (10%)	14 (18%)	1.94	0.95, 3.92	0.06
Absent	294 (88%)	230 (90%)	64 (82%)			
**Preterm Birth**						
Present	32 (9.2%)	23 (8.7%)	9 (11%)	1.4	0.19, 6.81	0.5
Absent	315 (91%)	242 (91%)	73 (89%)			
**SGA**						
SGA	69 (21%)	48 (19%)	21 (27%)	1.59	0.88, 2.88	0.1
Not SGA	265 (79%)	208 (81%)	57 (73%)			

SGA: small-for-gestational-age

## Data Availability

Data is available upon request from corresponding author.

## Abbreviations

SARS-CoV-2: severe acute respiratory syndrome coronavirus 2
HIV: human immune-deficiency virus
WLWH: women living with HIV
SSA: Sub-Saharan Africa
ANC: Antenatal care
ART: Anti-retroviral therapy
ENND: early neonatal death
LBW: Low birth weight
SGA: Small for gestational age
WHO: World Health Organization
OR: Odds Ratio
CI: Confidence Interval
aOR: adjusted odds ratio
