# Supplementary material for: Adverse Birth Outcomes Associated with SARS-CoV-2 Infection Among Pregnant Women with and without HIV: A Longitudinal Cohort Study
Source: J Acquir Immune Defic Syndr. Author manuscript; Available in PMC 2026 Nov 1. (PMC13686639; doi:10.1097/QAI.0000000000003924)
Supplement: Supplementary file 1 [file NIHMS2214995-supplement-supplemental_figures_and_tables.docx]

**Supplementary Materials**

**Supplemental Figure 1** – Directed Acyclic Graph

| **Supplemental Table 1a:** Association between population characteristics and low birth weight delivery | | | | |
| --- | --- | --- | --- | --- |
| **Characteristic** | **Overall**  N = 878*^1^* | **Normal Birth Weight**  N = 798*^1^* | **Low Birth Weight**  N = 80*^1^* | **p-value***^2^* |
| **Site** |  |  |  | 0.021 |
| Bvumbwe | 393 (45%) | 367 (46%) | 26 (33%) |  |
| Ndirande | 485 (55%) | 431 (54%) | 54 (68%) |  |
| **Maternal Age** | 28 (6) | 28 (6) | 29 (7) | 0.2 |
| **Marital Status** |  |  |  | 0.5 |
| Married | 796 (91%) | 726 (91%) | 70 (89%) |  |
| Not Married | 81 (9.2%) | 72 (9.0%) | 9 (11%) |  |
| **Educational Level** |  |  |  | 0.5 |
| Below Secondary | 445 (51%) | 402 (50%) | 43 (54%) |  |
| Secondary and above | 432 (49%) | 396 (50%) | 36 (46%) |  |
| **Gravidity** |  |  |  | 0.065 |
| Multigravida | 761 (87%) | 697 (87%) | 64 (80%) |  |
| Primigravida | 117 (13%) | 101 (13%) | 16 (20%) |  |
| **Parity** |  |  |  | 0.024 |
| Multiparity | 754 (86%) | 692 (87%) | 62 (78%) |  |
| Primiparity | 124 (14%) | 106 (13%) | 18 (23%) |  |
| **HIV Status** |  |  |  | 0.004 |
| Negative | 452 (51%) | 423 (53%) | 29 (36%) |  |
| Positive | 426 (49%) | 375 (47%) | 51 (64%) |  |
| **CD4 Counts** |  |  |  | 0.9 |
| <350 cells/mm3 | 67 (16%) | 41 (15%) | 8 (17%) |  |
| >350 cells/mm3 | 345 (84%) | 238 (85%) | 39 (83%) |  |
| **Viral Load** |  |  |  | 0.4 |
| Detectable | 66 (15%) | 60 (16%) | 6 (12%) |  |
| Undetectable | 360 (85%) | 315 (84%) | 45 (88%) |  |
| **ART Regimen** |  |  |  | 0.8 |
| DTG | 297 (70%) | 261 (70%) | 36 (72%) |  |
| NNRTI | 126 (30%) | 112 (30%) | 14 (28%) |  |
| *^1^* n (%); Mean (SD), *^2^* Pearson’s Chi-squared test; Wilcoxon rank sum test  DTG: Dolutegravir, NNRTI: Non-nucleoside reverse transcriptase inhibitors | | | | |

**Supplemental Table 1b:** Association Between Population Characteristics and Any Adverse Effect

| **Composite Outcome** | **Overall** | **Absent** | **Present** | **p-value*^2^*** |
| --- | --- | --- | --- | --- |
|  | **N = 905*^1^*** | **N = 608*^1^*** | **N = 297*^1^*** |  |
| Site |  |  |  | 0.014 |
| Bvumbwe | 412 (46%) | 294 (48%) | 118 (40%) |  |
| Ndirande | 493 (54%) | 314 (52%) | 179 (60%) |  |
| MaternalAge |  |  |  | 0.039 |
| Median (Q1, Q3) | 28 (23, 33) | 27 (23, 33) | 29 (24, 35) |  |
| Marital Status |  |  |  | 0.5 |
| Married | 819 (91%) | 548 (90%) | 271 (92%) |  |
| Not Married | 85 (9%) | 60 (10%) | 25 (8%) |  |
| Educational Level |  |  |  | 0.6 |
| Below Secondary | 461 (51%) | 306 (50%) | 155 (52%) |  |
| Secondary and above | 443 (49%) | 302 (50%) | 141 (48%) |  |
| Gravidity |  |  |  | 0.5 |
| Multigravida | 785 (87%) | 531 (87%) | 254 (86%) |  |
| Primigravida | 120 (13%) | 77 (13%) | 43 (14%) |  |
| Parity |  |  |  | 0.2 |
| Multiparity | 777 (86%) | 528 (87%) | 249 (84%) |  |
| Primiparity | 128 (14%) | 80 (13%) | 48 (16%) |  |
| HIV Status |  |  |  | 0.024 |
| Negative | 457 (50%) | 323 (53%) | 134 (45%) |  |
| Positive | 448 (50%) | 285 (47%) | 163 (55%) |  |
| CD4 Count |  |  |  | 0.3 |
| <350 cells/mm^3 | 69 (16%) | 41 (15%) | 28 (19%) |  |
| >=350 cells/mm^3 | 359 (84%) | 238 (85%) | 121 (81%) |  |
| Viral Load ever |  |  |  | 0.8 |
| Ever Detectable | 86 (20%) | 55 (19%) | 31 (21%) |  |
| Never Detectable | 347 (80%) | 228 (81%) | 119 (79%) |  |
| ART Regimen |  |  |  | 0.6 |
| DTG | 315 (71%) | 203 (72%) | 112 (69%) |  |
| NNRTI | 130 (29%) | 80 (28%) | 50 (31%) |  |
| *^1^* n (%) *, 2 Pearson’s Chi-squared test; Wilcoxon rank sum test*  DTG: Dolutegravir, NNRTI: Non-nucleoside reverse transcriptase inhibitors | | | | |

**Supplemental Table 1c:** Association between population characteristics and preterm birth

| **Preterm** | **Overall** | **Term** | **Preterm** | **p-value***^2^* |
| --- | --- | --- | --- | --- |
|  | N = 905*^1^* | N = 826*^1^* | N = 79*^1^* |  |
| **Site** |  |  |  | 0.8 |
| Bvumbwe | 412 (46%) | 377 (46%) | 35 (44%) |  |
| Ndirande | 493 (54%) | 449 (54%) | 44 (56%) |  |
| **Maternal age** |  |  |  | 0.5 |
| Median (Q1, Q3) | 28 (23, 33) | 28 (23, 33) | 28 (23, 35) |  |
| **Marital Status** |  |  |  | 0.3 |
| Married | 819 (91%) | 750 (91%) | 69 (87%) |  |
| Not Married | 85 (9.4%) | 75 (9.1%) | 10 (13%) |  |
| **Educational Level** |  |  |  | 0.9 |
| Below Secondary | 461 (51%) | 420 (51%) | 41 (52%) |  |
| Secondary and Above | 443 (49%) | 405 (49%) | 38 (48%) |  |
| **Gravidity** |  |  |  | 0.6 |
| Multigravida | 785 (87%) | 715 (87%) | 70 (89%) |  |
| Primigravida | 120 (13%) | 111 (13%) | 9 (11%) |  |
| **Parity** |  |  |  | 0.3 |
| Multiparity | 777 (86%) | 712 (86%) | 65 (82%) |  |
| Primiparity | 128 (14%) | 114 (14%) | 14 (18%) |  |
| **HIV Status** |  |  |  | 0.036 |
| Negative | 457 (50%) | 426 (52%) | 31 (39%) |  |
| Positive | 448 (50%) | 400 (48%) | 48 (61%) |  |
| **CD4 count** |  |  |  | 0.5 |
| <350 cells/mm^3 | 69 (16%) | 64 (16%) | 5 (13%) |  |
| >=350 cells/mm^3 | 359 (84%) | 324 (84%) | 35 (88%) |  |
| **Viral Load** |  |  |  | >0.9 |
| Ever Detectable | 86 (20%) | 78 (20%) | 8 (20%) |  |
| Never Detectable | 347 (80%) | 315 (80%) | 32 (80%) |  |
| **ART Regimen** |  |  |  | 0.5 |
| DTG | 315 (71%) | 279 (70%) | 36 (75%) |  |
| NNRTI | 130 (29%) | 118 (30%) | 12 (25%) |  |
| *^1^* n (%), ^2^ Pearson’s Chi-squared test; Wilcoxon rank sum test; Fisher’s exact test  DTG: Dolutegravir, NNRTI: Non-nucleoside reverse transcriptase inhibitors | | | | |

**Supplemental Table 1d:** Association between population characteristics and Small-for-gestational-age (SGA)

| **SGA** | **Overall** | **Not SGA** | **SGA** | **p-value***^2^* |
| --- | --- | --- | --- | --- |
|  | N = 877*^1^* | N = 709*^1^* | N = 168*^1^* |  |
| **Site** |  |  |  | **0.005** |
| Bvumbwe | 393 (45%) | 334 (47%) | 59 (35%) |  |
| Ndirande | 484 (55%) | 375 (53%) | 109 (65%) |  |
| **Maternal age** |  |  |  | 0.9 |
| Median (Q1, Q3) | 28 (23, 33) | 28 (23, 33) | 28 (23, 34) |  |
| **Marital Status** |  |  |  | 0.3 |
| Married | 795 (91%) | 640 (90%) | 155 (93%) |  |
| Not Married | 81 (9%) | 69 (10%) | 12 (7.2%) |  |
| **Educational Level** |  |  |  | 0.9 |
| Below Secondary | 445 (51%) | 361 (51%) | 84 (50%) |  |
| Secondary and Above | 431 (49%) | 348 (49%) | 83 (50%) |  |
| **Gravidity** |  |  |  | **0.1** |
| Multigravida | 760 (87%) | 621 (88%) | 139 (83%) |  |
| Primigravida | 117 (13%) | 88 (12%) | 29 (17%) |  |
| **Parity** |  |  |  | **0.12** |
| Multiparity | 753 (86%) | 615 (87%) | 138 (82%) |  |
| Primiparity | 124 (14%) | 94 (13%) | 30 (18%) |  |
| **HIV Status** |  |  |  | 0.3 |
| Negative | 452 (52%) | 371 (52%) | 81 (48%) |  |
| Positive | 425 (48%) | 338 (48%) | 87 (52%) |  |
| **CD4 count** |  |  |  | 0.4 |
| <350 cells/mm^3 | 67 (16%) | 49 (15%) | 18 (21%) |  |
| >=350 cells/mm^3 | 345 (84%) \| | 279 (85%) | 66 (79%) |  |
| **Viral Load** |  |  |  | 0.9 |
| Ever Detectable | 81 (20%) | 65 (20%) | 16 (19%) |  |
| Never Detectable | 334 (80%) | 265 (80%) | 69 (81%) |  |
| **ART Regimen** |  |  |  | 0.6 |
| DTG | 297 (70%) | 238 (71%) | 59 (68%) |  |
| NNRTI | 125 (30%) | 97 (29%) | 28 (32%) |  |
| *^1^* n (%)*, 2 Pearson’s Chi-squared test; Wilcoxon rank sum test* | | | | |

DTG: Dolutegravir, NNRTI: Non-nucleoside reverse transcriptase inhibitors

| **Supplemental Table 2a:** Characteristic of Sensitivity Cohort by SARS-CoV-2 Status, sensitivity cohort (enrolled >= April 1, 2020) | | | | |
| --- | --- | --- | --- | --- |
| **Characteristic** | **Overall** | **SARS-CoV-2 Negative** | **SARS-CoV-2 Positive** | **p-value***^2^* |
|  | N = 709*^1^* | N = 463*^1^* | N = 246*^1^* |  |
| **Site** |  |  |  | 0.19 |
| Bvumbwe | 354 (50%) | 240 (52%) | 114 (46%) |  |
| Ndirande | 355 (50%) | 223 (48%) | 132 (54%) |  |
| **Maternal Age** |  |  |  | 0.36 |
| Mean (SD) | 28 (6) | 28 (6) | 28 (7) |  |
| **Marital Status** |  |  |  | 0.77 |
| Married | 634 (90%) | 413 (89%) | 221 (90%) |  |
| Not Married | 74 (10%) | 50 (11%) | 24 (9.8%) |  |
| **Educational Level** |  |  |  | 0.63 |
| Below Secondary | 357 (50%) | 237 (51%) | 120 (49%) |  |
| Secondary and Above | 351 (50%) | 226 (49%) | 125 (51%) |  |
| **Gravidity** |  |  |  | >0.99 |
| Multigravida | 603 (85%) | 394 (85%) | 209 (85%) |  |
| Primigravida | 106 (15%) | 69 (15%) | 37 (15%) |  |
| **Delivery Method** |  |  |  | 0.64 |
| Others | 49 (6.9%) | 34 (7.3%) | 15 (6.1%) |  |
| Vaginal | 660 (93%) | 429 (93%) | 231 (94%) |  |
| **Infant Sex** |  |  |  | 0.74 |
| Female | 347 (49%) | 229 (50%) | 118 (48%) |  |
| Male | 361 (51%) | 233 (50%) | 128 (52%) |  |
| **HIV Status** |  |  |  | 0.54 |
| Negative | 420 (59%) | 270 (58%) | 150 (61%) |  |
| Positive | 289 (41%) | 193 (42%) | 96 (39%) |  |
| **CD4 Counts** |  |  |  | 0.022 |
| Mean (SD) | 591 (244) | 567 (238) | 639 (252) |  |
| **Viral Load** |  |  |  | 0.97 |
| Undetectable | 233 (84%) | 155 (84%) | 78 (85%) |  |
| Detectable | 44 (16%) | 30 (16%) | 14 (15%) |  |
| *^1^* n (%); Mean (SD)*^,^ 2 Pearson’s Chi-squared test; Wilcoxon rank sum test* | | | | |

| **Supplemental Table 2b:** Pregnancy outcomes by SARS-CoV-2 exposure, sensitivity cohort (enrolled >= April 1, 2020) | | | | |
| --- | --- | --- | --- | --- |
| **Characteristic** | **Overall** | **SARS-CoV-2 Negative** | **SARS-CoV-2 Positive** | **p-value***^2^* |
|  | N = 709*^1^* | N = 463*^1^* | N = 246*^1^* |  |
| **Any adverse outcome** |  |  |  | 0.28 |
| Present | 228 (32%) | 142 (31%) | 86 (35%) |  |
| Absent | 481 (68%) | 321 (69%) | 160 (65%) |  |
| **Low Birth Weight** |  |  |  | 0.83 |
| Present | 63 (9.1%) | 40 (8.8%) | 23 (9.7%) |  |
| Absent | 627 (91%) | 412 (91%) | 215 (90%) |  |
| **Small for Gestational Age** |  |  |  | 0.092 |
| Absent | 558 (81%) | 374 (83%) | 184 (77%) |  |
| Present | 131 (19%) | 77 (17%) | 54 (23%) |  |
| **Hospitalization** |  |  |  | 0.5 |
| Present | 57 (8.1%) | 40 (8.7%) | 17 (6.9%) |  |
| Absent | 651 (92%) | 422 (91%) | 229 (93%) |  |
| **Preterm Birth** |  |  |  | 0.77 |
| Present | 65 (9.2%) | 44 (9.5%) | 21 (8.5%) |  |
| Absent | 644 (91%) | 419 (90%) | 225 (91%) |  |
| **Early neonatal death/stillbirth** |  |  |  | 0.11 |
| Present | 16 (2.3%) | 14 (3.0%) | 2 (0.8%) |  |
| Absent | 693 (98%) | 449 (97%) | 244 (99%) |  |
| *^1^* n (%); *2 Pearson’s Chi-squared test; Wilcoxon rank sum test* | | | | |

| **Supplemental Table 2c:** Association between SARS-COV-2 Exposure and Pregnancy Outcomes from Logistic Regression Models Sensitivity Cohort (enrolled >= April 1, 2020) | | | | | | | | | |
| --- | --- | --- | --- | --- | --- | --- | --- | --- | --- |
| Outcome | Overall | SARS-CoV-2 Negative | SARS-CoV-2 Positive | OR | 95% CI | p-value | aOR | 95% CI | p-value |
| **Total Population** |  |  |  |  |  |  |  |  |  |
| **Any Adverse Event** |  |  |  |  |  |  |  |  |  |
| Present | 228 (32%) | 142 (31%) | 86 (35%) | 1.22 | 0.87, 1.69 | 0.2 | 1.19 | 0.85, 1.65 | 0.3 |
| Absent | 481 (68%) | 321 (69%) | 160 (65%) |  |  |  |  |  |  |
| **Low Birth Weight** |  |  |  |  |  |  |  |  |  |
| Present | 63 (9%) | 40 (9%) | 23 (10%) | 1.1 | 0.63, 1.87 | 0.7 | 1.06 | 0.61, 1.81 | 0.8 |
| Absent | 627 (91%) | 412 (91%) | 215 (90%) |  |  |  |  |  |  |
| **Preterm Birth** |  |  |  |  |  |  |  |  |  |
| Present | 65 (9%) | 44 (10%) | 21 (9%) | 0.89 | 0.51, 1.51 | 0.7 | 0.89 | 0.51, 1.51 | 0.7 |
| Absent | 644 (91%) | 419 (90%) | 225 (91%) |  |  |  |  |  |  |
| **Small for Gestational Age** |  |  |  |  |  |  |  |  |  |
| Present | 131 (19%) | 77 (17%) | 54 (23%) | 1.43 | 0.96, 2.10 | 0.07 | 1.38 | 0.93, 2.05 | 0.07 |
| Absent | 558 (81%) | 374 (83%) | 184 (77%) |  |  |  |  |  |  |
| **Women without HIV** |  |  |  |  |  |  |  |  |  |
| **Any Adverse Event** |  |  |  |  |  |  |  |  |  |
| Present | 126 (30%) | 77 (29%) | 49 (33%) | 1.22 | 0.79, 1.87 | 0.4 | 1.19 | 0.76, 1.83 | 0.4 |
| Absent | 294 (70%) | 193 (71%) | 101 (67%) |  |  |  |  |  |  |
| **Low Birth Weight** |  |  |  |  |  |  |  |  |  |
| Present | 27 (7%) | 19 (7%) | 8 (5%) | 0.75 | 0.30, 1.69 | 0.5 | 0.71 | 0.29, 1.64 | 0.4 |
| Absent | 388 (93%) | 248 (93%) | 140 (95%) |  |  |  |  |  |  |
| **Preterm Birth** |  |  |  |  |  |  |  |  |  |
| Present | 30 (7%) | 20 (7%) | 10 (7%) | 0.89 | 0.39, 1.92 | 0.8 | 0.91 | 0.40, 1.95 | 0.8 |
| Absent | 390 (93%) | 250 (93%) | 140 (93%) |  |  |  |  |  |  |
| **Small for Gestational Age** |  |  |  |  |  |  |  |  |  |
| Present | 76 (18%) | 44 (16%) | 32 (22%) | 1.4 | 0.84, 2.32 | 0.2 | 1.36 | 0.81, 2.28 | 0.2 |
| Absent | 339 (82%) | 223 (84%) | 116 (78%) |  |  |  |  |  |  |
| **Women Living with HIV** |  |  |  |  |  |  |  |  |  |
| **Any Adverse Event** |  |  |  |  |  |  |  |  |  |
| Present | 102 (35%) | 65 (34%) | 37 (39%) | 1.23 | 0.74, 2.05 | 0.4 | 1.22 | 0.73, 2.04 | 0.4 |
| Absent | 187 (65%) | 128 (66%) | 59 (61%) |  |  |  |  |  |  |
| **Low Birth Weight** |  |  |  |  |  |  |  |  |  |
| Present | 36 (13%) | 21 (11%) | 15 (17%) | 1.56 | 0.75, 3.18 | 0.2 | 1.53 | 0.73, 3.13 | 0.2 |
| Absent | 239 (87%) | 164 (89%) | 75 (83%) |  |  |  |  |  |  |
| **Preterm Birth** |  |  |  |  |  |  |  |  |  |
| Present | 35 (12%) | 24 (12%) | 11 (11%) | 0.91 | 0.41, 1.91 | 0.8 | 0.89 | 0.40, 1.87 | 0.8 |
| Absent | 254 (88%) | 169 (88%) | 85 (89%) |  |  |  |  |  |  |
| **Small for Gestational Age** |  |  |  |  |  |  |  |  |  |
| Present | 55 (20%) | 33 (18%) | 22 (24%) | 1.48 | 0.80, 2.72 | 0.2 | 1.48 | 0.79, 2.72 | 0.2 |
| Absent | 219 (80%) | 151 (82%) | 68 (76%) |  |  |  |  |  |  |

| Supplemental Table 2d: SARS-CoV Infection and Pregnancy Outcome Among Women Living with HIV by Viral Load Status, Sensitivity Cohort (enrolled >= April 1, 2020) | | | | | | |  |  |
| --- | --- | --- | --- | --- | --- | --- | --- | --- |
| **All HIV-Positive** | **Overall** | **SARS-CoV-2 Negative** | **SARS-CoV-2 Positive** | **OR** | **95% CI** | **p-value** |  |  |
| **Any Adverse Event** |  |  |  |  |  |  |  |  |
| Present | 102 (35%) | 65 (34%) | 37 (39%) | 1.23 | 0.74, 2.05 | 0.4 |  |  |
| Absent | 187 (65%) | 128 (66%) | 59 (61%) |  |  |  |  |  |
| **Low Birth Weight** |  |  |  |  |  |  |  |  |
| Present | 36 (13%) | 21 (11%) | 15 (17%) | 1.56 | 0.75, 3.18 | 0.2 |  |  |
| Absent | 239 (87%) | 164 (89%) | 75 (83%) |  |  |  |  |  |
| **Preterm Birth** |  |  |  |  |  |  |  |  |
| Present | 35 (12%) | 24 (12%) | 11 (11%) | 0.91 | 0.41, 1.91 | 0.8 |  |  |
| Absent | 254 (88%) | 169 (88%) | 85 (89%) |  |  |  |  |  |
| **SGA** |  |  |  |  |  |  |  |  |
| Present | 55 (20%) | 33 (18%) | 22 (24%) | 1.48 | 0.80, 2.72 | 0.2 |  |  |
| Absent | 219 (80%) | 151 (82%) | 68 (76%) |  |  |  |  |  |
| **Detectable Viral Load** |  |  |  |  |  |  |  |  |
| **Any Adverse Event** |  |  |  |  |  |  |  |  |
| Present | 13 (30%) | 8 (27%) | 5 (36%) | 1.51 | 0.30, 7.14 | 0.7 |  |  |
| Absent | 31 (70%) | 22 (73%) | 9 (64%) |  |  |  |  |  |
| **Low Birth Weight** |  |  |  |  |  |  |  |  |
| Present | 3 (7%) | 2 (7%) | 1 (9%) | 1.39 | 0.02, 29.48 | 0.9 |  |  |
| Absent | 38 (93%) | 28 (93%) | 10 (91%) |  |  |  |  |  |
| **Preterm Birth** |  |  |  |  |  |  |  |  |
| Present | 5 (11%) | 3 (10%) | 2 (14%) | 1.49 | 0.11, 14.81 | 0.7 |  |  |
| Absent | 39 (89%) | 27 (90%) | 12 (86%) |  |  |  |  |  |
| **SGA** |  |  |  |  |  |  |  |  |
| Present | 6 (15%) | 4 (13%) | 2 (18%) | 1.43 | 0.11, 12.13 | 0.7 |  |  |
| Absent | 35 (85%) | 26 (87%) | 9 (82%) |  |  |  |  |  |
| **Undetectable Viral Load** |  |  |  |  |  |  |  |  |
| **Any Adverse Event** |  |  |  |  |  |  |  |  |
| Present | 79 (34%) | 49 (32%) | 30 (38%) | 1.35 | 0.76, 2.38 | 0.3 |  |  |
| Absent | 154 (66%) | 106 (68%) | 48 (62%) |  |  |  |  |  |
| **Low Birth Weight** |  |  |  |  |  |  |  |  |
| Present | 30 (13%) | 17 (11%) | 13 (17%) | 1.65 | 0.74, 3.60 | 0.2 |  |  |
| Absent | 196 (87%) | 134 (89%) | 62 (83%) |  |  |  |  |  |
| **Preterm Birth** |  |  |  |  |  |  |  |  |
| Present | 23 (10%) | 15 (10%) | 8 (10%) | 1.07 | 0.41, 2.58 | 0.9 |  |  |
| Absent | 210 (90%) | 140 (90%) | 70 (90%) |  |  |  |  |  |
| **SGA** |  |  |  |  |  |  |  |  |
| Present | 48 (21%) | 29 (19%) | 19 (25%) | 1.43 | 0.73, 2.75 | 0.3 |  |  |
| Absent | 178 (79%) | 122 (81%) | 56 (75%) |  |  |  |  |  |
| SGA: small-for-gestational-age | |  |  |  |  | | |  |
