# Supplementary material for: Adverse Birth Outcomes Associated With SARS-CoV-2 Infection Among Pregnant Women With and Without HIV: A Longitudinal Cohort Study
Source: J Acquir Immune Defic Syndr. 2026 Jul 27;101(11):1259–68. doi: 10.1097/QAI.0000000000003924 (PMC13686639; doi:10.1097/QAI.0000000000003924)
Supplement: Supplementary file 1 [file qai-101-1259-s001.pdf]

Table S1. Unadjusted and adjusted effect of placental viral load, peripheral viral load, and CD4+ T-cell count on prevalence of low birthweight and excluding observations with PTD, stratified by maternal malaria status**.**

	Malaria-positive				Malaria-negative			
METRIC	uPR	95% CI	aPR	95% CI	uPR	95% CI	aPR	95% CI
**Placental viral load**								
Per 1·0-log_{10} increase	1·49	0·88, 2·52	1·64	0·98, 2·74	1·30	0·93, 1·83	1·27	0·94, 1·73
**Peripheral viral load**								
Per 1·0-log_{10} increase	1·64	0·83, 3·22	1·64	0·81, 3·30	1·43	0·99, 2·06	1·46	1·00, 2·13
**CD4+ T-cell count**								
Per 100-cell/µl decrease	1·03	0·84, 1·26	1·03	0·85, 1·24	1·14	1·02, 1·28	1·15	1·02, 1·28

uPR: unadjusted prevalence ratio
aPR: adjusted prevalence ratio; all adjusted models control for residence, maternal education, primigravid status and maternal anemia
CI: confidence interval

Table S2. Unadjusted and adjusted effect of placental viral load, peripheral viral load, and CD4+ T-cell count on prevalence of low birthweight and excluding observations with HIV-positive infants (HIV-infected in utero), stratified by maternal malaria status.

	Malaria-positive				Malaria-negative			
METRIC	uPR	95% CI	aPR	95% CI	uPR	95% CI	aPR	95% CI
**Placental viral load**								
Per 1·0-$\log_{10}$ increase	1·13	0·71, 1·79	1·18	0·73, 1·91	1·34	0·99, 1·80	1·37	1·04, 1·81
**Peripheral viral load**								
Per 1·0-$\log_{10}$ increase	0·72	0·46, 1·13	0·72	0·46, 1·13	1·51	1·14, 2·01	1·49	1·12, 1·97
**CD4+ T-cell count**								
Per 100-cell/µl decrease	1·00	0·85, 1·17	1·00	0·86, 1·16	1·08	0·99, 1·18	1·08	0·99, 1·18

uPR: unadjusted prevalence ratio
aPR: adjusted prevalence ratio; all adjusted models control for residence, maternal education, primigravid status and maternal anemia
CI: confidence interval

Table S3. Unadjusted and adjusted effect of placental viral load, peripheral viral load, and CD4+ T-cell count on prevalence of preterm delivery and excluding observations with HIV-positive infants (HIV-infected in utero), stratified by maternal malaria status**.**

	Malaria-positive				Malaria-negative			
METRIC	uPR	95% CI	aPR	95% CI	uPR	95% CI	aPR	95% CI
**Placental viral load**								
Per 1·0-log_{10} increase	1·07	0·67, 1·70	1·01	0·63, 1·61	1·25	0·90, 1·74	1·24	0·90, 1·71
**Peripheral viral load**								
Per 1·0-log_{10} increase	0·63	0·43, 0·92	0·53	0·34, 0·81	1·15	0·84, 1·57	1·11	0·81, 1·53
**CD4+ T-cell count**								
Per 100-cell/µl decrease	1·07	0·91, 1·25	1·05	0·90, 1·22	1·19	1·06, 1·34	1·18	1·05, 1·32

uPR: unadjusted prevalence ratio
aPR: adjusted prevalence ratio; all adjusted models control for residence, maternal education, primigravid status and maternal anemia
CI: confidence interval

Table S4. Unadjusted and adjusted effect of placental viral load, peripheral viral load, and CD4+ T-cell count on prevalence of preterm delivery, stratified by diagnostic method for maternal malaria.

	Peripheral blood smear-positive				Peripheral blood smear-negative			
	uPR	95% CI	aPR	95% CI	uPR	95% CI	aPR	95% CI
**Placental viral load**								
Per 1·0-log_{10} increase	1·27	0·48, 3·38	1·89	0·41, 4·07	1·29	1·05, 1·59	1·24	1·01, 1·52
**Peripheral viral load**								
Per 1·0-log_{10} increase	0·52	0·30, 0·89	0·46	0·27, 0·78	1·14	0·89, 1·46	1·06	0·85, 1·32
**CD4+ T-cell count**								
Per 100-cell/µl decrease	1·00	0·71, 1·39	0·92	0·67, 1·25	1·15	1·06, 1·25	1·14	1·04, 1·24
	Placental blood-positive				Placental blood-negative			
	uPR	95% CI	aPR	95% CI	uPR	95% CI	aPR	95% CI
**Placental viral load**								
Per 1·0-log_{10} increase	0·98	0·54, 1·79	0·95	0·53, 1·68	1·30	1·04, 1·61	1·27	1·02, 1·58
**Peripheral viral load**								
Per 1·0-log_{10} increase	0·52	0·26, 1·03	0·57	0·28, 1·15	1·17	0·91, 1·49	1·08	0·86, 1·36
**CD4+ T-cell count**								
Per 100-cell/µl decrease	1·12	0·85, 1·48	1·10	0·83, 1·45	1·14	1·04, 1·24	1·12	1·02, 1·22
	Histology-positive				Histology-negative			
	uPR	95% CI	aPR	95% CI	uPR	95% CI	aPR	95% CI
**Placental viral load**								
Per 1·0-log_{10} increase	1·05	0·69, 1·59	1·02	0·68, 1·55	1·33	1·03, 1·71	1·30	1·02, 1·66
**Peripheral viral load**								
Per 1·0-log_{10} increase	0·70	0·48, 1·04	0·57	0·36, 0·91	1·15	0·86, 1·54	1·09	0·82, 1·46
**CD4+ T-cell count**								
Per 100-cell/µl decrease	1·06	0·90, 1·24	1·04	0·90, 1·22	1·13	1·03, 1·25	1·12	1·01, 1·23

uPR: unadjusted prevalence ratio
aPR: adjusted prevalence ratio; all adjusted models control for residence, maternal education, primigravid status and maternal anemia
CI: confidence interval

Table S5. Unadjusted and adjusted effect of placental viral load, peripheral viral load, and CD4+ T-cell count on prevalence of low birthweight, stratified by diagnostic method for maternal malaria.

	**Peripheral blood smear-positive**				**Peripheral blood smear-negative**			
	**uPR**	**95% CI**	**aPR**	**95% CI**	**uPR**	**95% CI**	**aPR**	**95% CI**
**Placental viral load**								
Per 1·0-$\log_{10}$ increase	1·23	0·60, 2·50	1·39	0·73, 2·65	1·24	1·03, 1·49	1·20	1·01, 1·43
**Peripheral viral load**								
Per 1·0-$\log_{10}$ increase	0·98	0·42, 2·28	0·98	0·41, 2·33	1·32	1·06, 1·64	1·28	1·03, 1·59
**CD4+ T-cell count**								
Per 100-cell/µl decrease	0·99	0·68, 1·46	0·98	0·68, 1·42	1·10	1·03, 1·18	1·09	1·02, 1·17
	**Placental blood-positive**				**Placental blood-negative**			
	**uPR**	**95% CI**	**aPR**	**95% CI**	**uPR**	**95% CI**	**aPR**	**95% CI**
**Placental viral load**								
Per 1·0-$\log_{10}$ increase	1·15	0·65, 2·05	1·12	0·62, 2·01	**1·23**	1·01, 1·50	1·26	1·05, 1·52
**Peripheral viral load**								
Per 1·0-$\log_{10}$ increase	0·64	0·33, 1·24	0·75	0·40, 1·43	**1·37**	1·09, 1·72	1·23	1·00, 1·51
**CD4+ T-cell count**								
Per 100-cell/µl decrease	1·03	0·79, 1·33	0·98	0·74, 1·29	**1·08**	1·01, 1·16	1·08	1·01, 1·16
	**Histology-positive**				**Histology-negative**			
	**uPR**	**95% CI**	**aPR**	**95% CI**	**uPR**	**95% CI**	**aPR**	**95% CI**
**Placental viral load**								
Per 1·0-$\log_{10}$ increase	1·16	0·78, 1·72	1·20	0·79, 1·81	1·21	0·98, 1·51	1·23	1·01, 1·50
**Peripheral viral load**								
Per 1·0-$\log_{10}$ increase	0·76	0·52, 1·13	0·77	0·49, 1·20	1·42	1·10, 1·83	1·26	1·01, 1·56
**CD4+ T-cell count**								
Per 100-cell/µl decrease	0·94	0·84, 1·07	0·95	0·85, 1·06	1·09	1·01, 1·18	1·09	1·00, 1·17

uPR: unadjusted prevalence ratio
aPR: adjusted prevalence ratio; all adjusted models control for residence, maternal education, primigravid status and maternal anemia
CI: confidence interval
